# Spatial and Temporal Variations of Satellite-Derived Multi-Year Particulate Data of Saudi Arabia: An Exploratory Analysis

**DOI:** 10.3390/ijerph111111152

**Published:** 2014-10-27

**Authors:** Yusuf A. Aina, Johannes H. van der Merwe, Habib M. Alshuwaikhat

**Affiliations:** 1 Department of Geomatics Engineering Technology, Yanbu Industrial College, Yanbu 41912, Saudi Arabia; 2 Department of Geography and Environmental Studies, University of Stellenbosch, Stellenbosch 7600, South Africa; E-Mail: jhvdm@sun.ac.za; 3 Department of City and Regional Planning, King Fahd University of Petroleum and Minerals, Dhahran 31261, Saudi Arabia; E-Mail: habibms@kfupm.edu.sa

**Keywords:** satellite data, fine particulate matter, air pollution, geographic information system, health risks, spatial analysis, Saudi Arabia

## Abstract

The effects of concentrations of fine particulate matter on urban populations have been gaining attention because fine particulate matter exposes the urban populace to health risks such as respiratory and cardiovascular diseases. Satellite-derived data, using aerosol optical depth (AOD), have been adopted to improve the monitoring of fine particulate matter. One of such data sources is the global multi-year PM_2.5_ data (2001–2010) released by the Center for International Earth Science Information Network (CIESIN). This paper explores the satellite-derived PM_2.5_ data of Saudi Arabia to highlight the trend of PM_2.5_ concentrations. It also examines the changes in PM_2.5_ concentrations in some urbanized areas of Saudi Arabia. Concentrations in major cities like Riyadh, Dammam, Jeddah, Makkah, Madinah and the industrial cities of Yanbu and Jubail are analyzed using cluster analysis. The health risks due to exposure of the populace are highlighted by using the World Health Organization (WHO) standard and targets. The results show a trend of increasing concentrations of PM_2.5_ in urban areas. Significant clusters of high values are found in the eastern and south-western part of the country. There is a need to explore this topic using images with higher spatial resolution and validate the data with ground observations to improve the analysis.

## 1. Introduction

### 1.1. Background

The continuous monitoring of particulate matter (PM) is very vital to achieving sound environmental and public health bearing in mind the adverse health impacts of these particles. Exposure to fine particulate matter (PM_2.5_) can cause health problems such as asthma, bronchitis, lung inflammation and other respiratory and cardiovascular diseases. Thus, the exposure of a population to particulates could result in increased hospital visits and mortality and thereby have negative impacts on social and environmental sustainability. Particulate matter monitoring is becoming more compelling due to the increasing levels of emissions of the particles from both natural and human-induced sources. This is particularly important for countries with high levels of PM concentrations. The issue of increasing levels of emissions/concentrations of PM has attained international dimension as, for example, smokes from forest fires in Indonesia impacted air quality in Malaysia and Singapore.

Conventional PM monitoring is based on ground measurements that cover limited areas because huge resources are needed to maintain a wide network of measuring devices. Remote sensing is currently being used to improve the effectiveness of particulate monitoring. Satellite imagery supplements conventional methods of data gathering and provides an opportunity for wide area coverage. Particulate matter is estimated from satellite images through the derivation of aerosol optical depth (AOD). There are established relationships between AOD and PM_2.5_ concentrations. The relationships are explored to make quantitative estimates of PM_2.5_ from satellite images such as the Moderate Resolution Imaging Spectroradiometer (MODIS). The particulate matter estimate derived from MODIS can be calibrated by using ground measurements to compute the correlation between satellite-derived data and field data. Such correlations are very useful in establishing satellite-based continuous monitoring of particulate matter.

In an effort to establish a global continuous monitoring of PM_2.5_, the Center for International Earth Science Information Network (CIESIN) produced a set of global annual average PM_2.5_ grids from MODIS and Multi-angle Imaging SpectroRadiometer (MISR), 2001 to 2010 [1]. The data sets cover the world from Latitude 70°N to Latitude 60°S and have a spatial resolution of approximately 50 km. Zell and Weber [2] used the data to compute country estimates of PM_2.5_ from 2002 to 2009. They computed 3-year moving averages for the countries and used CIESIN’s Global Rural-Urban Mapping Project (GRUMP) 1 km population grids to compute population-weighted PM_2.5_ exposures. The results have been adopted by Emerson *et al.* [3] to assess air quality in the 2012 Environmental Performance Index Report.

The results indicated that air quality in Saudi Arabia is getting worse as the population-weighted average PM_2.5_ values increased from 13.70 µg/m^3^ in 2002 to 15.11 µg/m^3^ in 2009. The results might be higher than that if the average particulate values are weighted by recent population data. The GRUMP data is either an estimate or old data that might not properly depict current population. Most of the studies on particulate matter in Saudi Arabia focused on PM_10_ and a few cities. Thus, this paper uses more recent population data to explore the changes in PM_2.5_ concentrations and exposures from 2002 to 2009 at the cities level. It also assesses changes in PM_2.5_ exposures in selected Saudi Arabian cities.

### 1.2. Particulate Matter, Health Risks and Remote Sensing

The issues of concentrations of particulate matter have generated some research interest due to the implications for sustainable development. Some studies [4,5,6,7,8,9] have highlighted the adverse impacts of high level of particulate matter concentrations on health. The health effects depend on the composition and size of the particles and the physiology of the exposed population. Zhou *et al.* [10] documented the presence of trace metals, which have adverse health implications, in atmospheric fine particles in an industrial city in China. Related to the issue of trace metals in particulate matter is the association between particulate matter concentrations and cancer, particularly lung cancer. Studies [11,12] showed a consistent association between particulate matter and lung cancer and the International Agency for Research on Cancer (IARC) has classified particulate matter as a carcinogenic pollutant [13]. Franck *et al.* [8] suggested in their study of the effect of particle size on heart-related disorders, that the smaller the particle sizes the worse the health effects of exposure. However, they noted that the effects of coarse particles (PM_10_) last longer than the effects of finer particles. A recent study by Son and Bell [6] highlighted the impacts of sub-daily exposures and concluded that exposures to PM_10_ were associated with cardiovascular mortality. They recommended 24 h averaging time as a metric for health research and regulations. However, remote sensing observations of PM are mainly suitable for monthly and yearly assessment. This is one of the challenges highlighted by Hoff and Christopher [14] as militating against using satellite measurements as the sole system for PM monitoring.

In order to improve the use of satellite for PM monitoring, some research articles on satellite measurement of PM values have focused on the calibration of PM/AOD relationship since the relationship varies across regions and seasons. For instance, Li *et al.* [15], Gupta and Christopher [16], Kumar *et al.* [17], Schaap *et al.* [18], Natunen *et al.* [19], Tian and Chen [20] and Lee *et al.* [21] have carried out calibration studies in Finland, China, India, The Netherlands, Canada and the United States. The studies confirmed the large spatial and temporal variations of PM/AOD relationship. The correlation coefficients ranged from 0.52 to 0.97. Hu found, by using 2003 and 2004 MODIS data of the United States, that PM/AOD correlation coefficients varied from 0.22 in the west to 0.67 in the east [5]. Moreover, AOD distribution varies with land use structure or topography [22]. Gupta *et al.* [23] presented a global study of PM/AOD relationship by assessing the values at 26 locations in Delhi, Hong Kong, New York, Switzerland and Sydney. They suggested that aerosol vertical distribution data could refine their analysis. Vertical profiles were included in the study by Van Donkelaar *et al.* [24], in which they presented a continuous surface of global estimates of fine particulate matter concentrations extended over 6 years (2001–2006). They noted that 80% of world population resides in areas where the World Health Organization (WHO) Air Quality Guide (AQG) of 10 µg/m^3^ is exceeded. Also, 50% of eastern Asian population resides in areas where the concentrations of fine particulate matter exceed WHO Air Quality Interim Target-3 of 35 µg/m^3^ [24]. Battelle Memorial Institute and CIESIN [1] improved the work of Van Donkelaar *et al.* [24] by using a faster algorithm for deriving PM_2.5_ data and extending the years of analysis to 2010.

In the context of Saudi Arabia, most studies have focused on measuring the concentrations and compositions of particulate matter in major cities [25,26,27,28,29] and assessing the impacts of major events such as Hajj (pilgrimage) and dust storms [30,31,32,33]. For example, Rushdi *et al.* [25] showed that PM concentrations (both PM_2.5_ and PM_10_) were higher in 2007 than 2006 and the mean concentrations in industrial and suburban areas are higher than that of other urban land use areas. Riyadh city centre was reported to have the lowest concentrations of particulate matter. Aburas *et al.* [26] and Khodeir *et al.* [27] determined that the elemental composition of PM_2.5_ in Jeddah in 2008, 2009 and 2009 included carcinogenic elements such as lead, nickel, vanadium and selenium. These elements could pose health risks for the population of the city. In a departure from the trend of using ground measurements, Othman *et al.* [31] developed a multispectral algorithm for generating PM_10_ concentrations from Landsat ETM+ imagery. They reported correlation coefficients that are greater than 0.8 in Makkah, Mina and Arafah. The PM_10_ concentration values were computed for Makkah and its neighbouring areas only. There is a need for more satellite-based study of particulate matter in Saudi Arabia, in order to improve the monitoring of particulates resulting from both natural and anthropogenic activities. The objectives of this study are to: (1) assess exposure to fine particulate matter in Saudi Arabia using satellite-derived PM_2.5_ values and recent population data, (2) analyze the exposure to PM_2.5_ for some selected Saudi Arabian cities using cluster analysis and (3) examine the differences in PM_2.5_ concentrations and exposures between Saudi industrial cities and other Saudi cities.

## 2. Materials and Methods

### 2.1. Study Area

**Figure 1:**
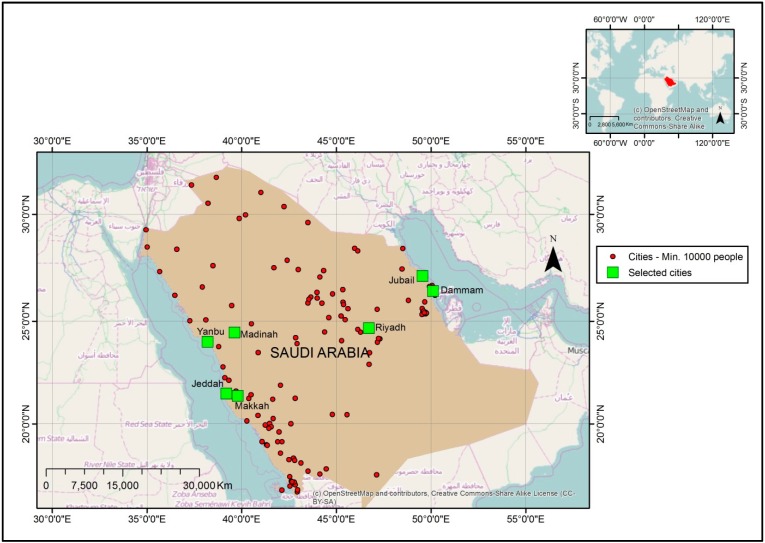
Location of selected cities.

This study focused on Saudi Arabia, one of the member countries of the Gulf Cooperation Council (GCC). Saudi Arabia occupies the major part of the Arabian Peninsula with an area of about 2 million km^2^ (Figure 1). The population of Saudi Arabia is about 27 million, according to the 2010 census. A high percentage of the population (more than 80%) lives in the cities. The analysis and illustrations in this study were carried out at three spatial levels. Data for the whole country was used for the first level while cities with population of 10,000 or more were selected for the second level and seven of these cities were selected for further analysis of fine particulate matter exposure at the third level. The seven cities (Riyadh, Dammam, Jeddah, Makkah, Madinah and the industrial cities of Yanbu and Jubail) (Figure 1) accommodate about 50% of the Saudi Arabian population. The concentration of the populace in few cities has implications for the sustainability of the cities [34] and the risk of exposure of a high percentage of the population to particulate matter. The cities of Riyadh, Dammam, Jeddah, Makkah and Madinah are not solely residential and commercial centres; they also have large industrial areas. For example, Riyadh and Dammam have about two industrial cities each. The industrial activities in these cities could lead to increases in exposure of the populace to particulates. The Saudi government has been making efforts to reduce the influx of population into these cities especially Riyadh. For instance, the industrial cities of Jubail and Yanbu were established as new towns to reduce the population pressure on existing cities and foster industrial development. However, due to Riyadh’s local and global status, it continues to attract a large population.

### 2.2. Population and PM_2.5_ Data

The satellite-derived PM_2.5_ was downloaded from CIESIN website [1]. The data, derived from MODIS/MISR AOD data, provides a multiyear (2001–2010) continuous surface of concentrations of PM_2.5_. The datasets were validated by using ground measurements from AErosol RObotic NETwork (AERONET) (which includes Saudi stations) and high levels of agreement were achieved [35]. The raster grids have a spatial resolution of about 50km. The PM_2.5_ values are in µg/m^3^ multiplied by a factor of 1000. Population data (according to 2004 and 2010 census) were acquired from the Central Department of Statistics and Information (CDSI), Saudi Arabia [36]. The data include lists of cities with a population of 5000 people or more. The cities with a population of 10,000 people or more were selected for the second spatial level of analysis. The cities accommodated about 79% and 80% of Saudi Arabian population in 2004 and 2010 respectively.

### 2.3. Methodology

The satellite-derived data layers (2001–2010) were clipped to the Saudi Arabia boundary to derive the PM_2.5_ concentrations over Saudi Arabia. The 3-year moving averages of the data (2002–2009) were also computed in ArcGIS 10.1. The Saudi Arabia population data from CDSI lacks location information. Thus, the location of each city (142 cities) was derived from Saudi Geological Survey publications [37] and Google Earth. After completing the inclusion of location data, the population data was converted to a shapefile in ArcGIS 10.1. The cities’ shapefile was used to extract PM_2.5_ values from the PM_2.5_ data layers so that each city will have a corresponding PM_2.5_ value. Three-year moving averages (2002–2009) were also computed for each city. The 50 km by 50 km cells of the data cover the urban extent of most of the cities except Riyadh and Jeddah that are covered by two cells. The averages of the two cells (covering each city) were computed to derive the values for Riyadh and Jeddah. After extracting the PM_2.5_ values of the cities, the population data was used to compute the population-weighted PM_2.5_ exposures values for each year. The PM_2.5_ population exposure is usually computed by calculating the average PM_2.5_ value for all the raster cells within a study area. Population weight can be applied to the average PM_2.5_ value to let areas with large population contribute more to the final result than other areas. The population-weighted PM_2.5_ exposure values were calculated by multiplying the PM_2.5_ of each cell (city) by its population and dividing the result by the total population of all the cells (cities). The sum of all the fractions (for each cell or city) was computed to derive the population-weighted PM2.5 exposure values.

After the derivation and computation of PM_2.5_ values and population-weighted exposures, cluster analysis was carried out to explore the geographical distributions and patterns of PM_2.5_ concentrations. The implementation of Anselin local Moran’s I statistic of spatial association [38] in ArcGIS 10.1, Cluster and Outlier Analysis tool, was used for exploratory analysis of the data. Given the input data and the required parameters, the tool computes a local Moran’s I value and clusters of features with high or low attribute values [38]. It also computes a z-score and a *p*-value that indicate whether one can reject or accept the null hypothesis (complete spatial randomness) [39]. It identifies the cluster types that are statistically significant; cluster of high values (HH), cluster of low values (LL), a high value that is surrounded by low values (HL), and a low value that is surrounded by high values (LH) [39]. The output of the analysis tool is sensitive to the given parameters especially the threshold distance. So, the threshold distance should be carefully chosen.

One way of identifying a suitable distance is to compute global Moran’s I values and z-scores for the data at varying distances by changing the threshold distance values. The peaks of the z-scores indicate distances where clustering processes are most pronounced [39]. For this study, the z-scores were calculated at every 5 km starting from 40 km to 400 km. The increment was changed to 50 km after the distance of 100 km. Two peaks were recorded at 40 km and 55 km but some points did not have neighbours at these distances, so the distance of 55 km was adopted with the “zone of indifference” option for conceptualization of spatial relationship. That is, neighbouring points within 55 km were given more weight in the analysis. The peaks for 2002 and 2009 data were the same and similar parameters were used in the analysis of data for both years. The two years were chosen to examine the changes that have occurred in PM_2.5_ concentrations in Saudi cities.

## 3. Results

**Figure 2:**
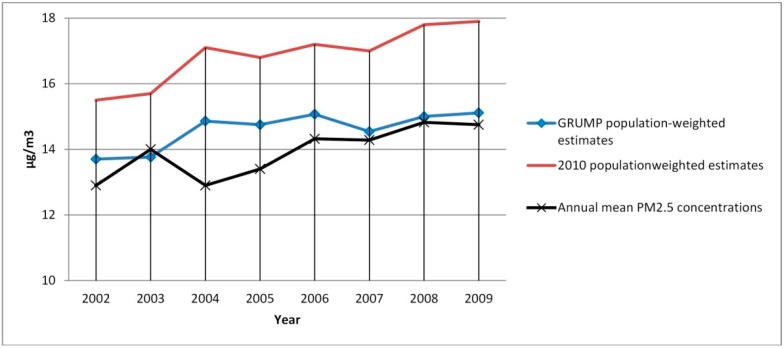
Population-weighted PM_2.5_ estimates (2002–2009).

**Figure 3:**
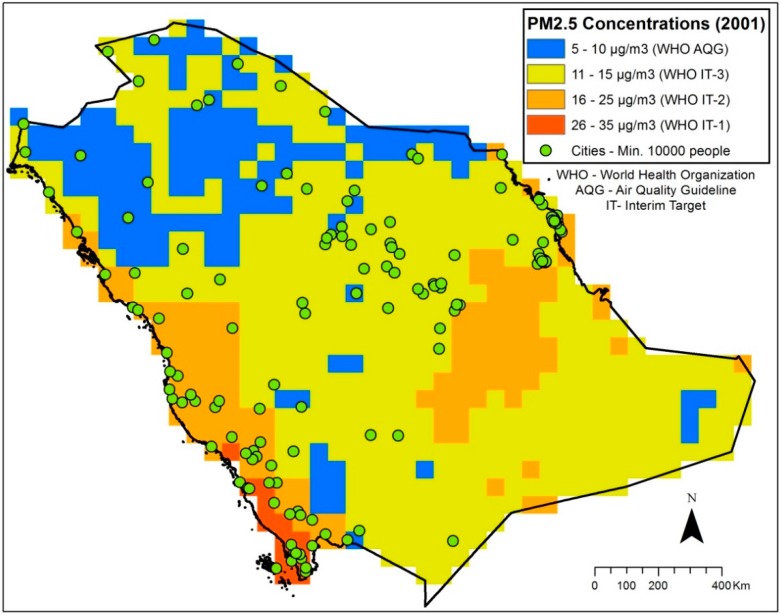
PM_2.5_ concentrations (un-weighted) in 2001.

Note: Data Source: Battelle Memorial Institute and CIESIN [1].

**Figure 4:**
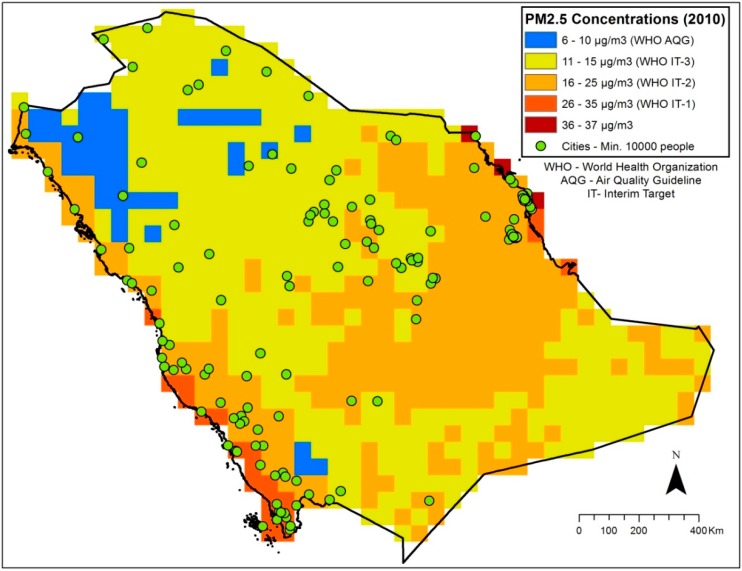
PM_2.5_ concentrations (un-weighted) in 2010.

Note: Data Source: Battelle Memorial Institute and CIESIN [1].

The results show an increasing trend in the annual mean concentrations of PM_2.5_ in Saudi Arabia (about 2 µg/m^3^ in seven years). The annual mean concentrations increased from 12.9 µg/m^3^ in 2002 to 14.8 µg/m^3^ in 2009 (Figure 2). The result of the population-weighted exposures shows that using recent population data gives values that are 2 µg/m^3^ higher than the GRUMP estimates. The range of the values is from 15.5 µg/m^3^ in 2002 to 17.9 µg/m^3^ in 2009 unlike the GRUMP estimates that range from 13.7 to 15.11 µg/m^3^ (Figure 2). The spatial distribution and patterns of the PM_2.5_ concentrations in 2001 and 2010 are shown in figures 3 and 4. The maps show the spatial distributions according to WHO air quality guidelines and targets [40]. The 2001 map shows that PM_2.5_ concentrations in Saudi Arabia does not exceed WHO Interim Target-1 (35 µg/m^3^) while the 2010 map shows that the concentrations have exceeded the Interim Target-1 (Figure 3 and Figure 4). Also, the area covered by WHO Interim Target-2 (25 µg/m^3^) has increased in 2010 while the area covered by Interim Target-3 (15 µg/m^3^) has reduced (Figure 3 and Figure 4).

The changes (2004–2010) in population exposure to PM_2.5_ concentrations are shown in Table 1 below. The table shows the number of cities, population and percentage of the population that are exposed to PM_2.5_ concentrations exceeding WHO targets. For example, the percentage of the population that is exposed to particulates exceeding 35 µg/m^3^ increased from 0% in 2004 to 8% in 2010. Also, the percentage of those who are exposed to concentrations that are more than 35 µg/m^3^ increased from 93% in 2004 to 95% in 2010. Table 2 shows the PM_2.5_ concentrations of selected cities with Madinah city having the only decreasing trend among the cities. The values for the industrial cities show that Yanbu has higher values than others except Dammam but the values for Jubail are not so high relative to other cities. However, Jubail has the second highest increase from 2002 to 2009. Some of the cities (Jeddah, Makkah, Dammam and Yanbu) have a break from the increasing trend in 2005. The table shows decreases in the values for Jeddah (from 21.1 to 20.3 µg/m^3^), Makkah (from 20.2 to 19.1 µg/m^3^), Dammam (from 33.4 to 30.3 µg/m^3^) and Yanbu (from 19.3 to 19.1 µg/m^3^) from 2004 to 2005. It can be noted from the table that an average of about 150,000 people are exposed to PM_2.5_ concentrations of about 20 µg/m^3^ in 2009.

**Table 1:** Changes in population exposure to PM_2.5_ concentrations (un-weighted) by WHO guidelines and targets.

Year: 2004 Total Population: 17,853,490 (79% of National Population)				Year: 2010 Total Population: 21,679,808 (80% of National Population)			
WHO Guideline and Targets (in µg/m^3^)	Number of Cities (Min. 10,000 people)	Population	%	WHO Guideline and Targets (in µg/m^3^)	Number of Cities (Min. 10,000 people)	Population	%
10	134	16,649,253	93	10	136	20,608,138	95
15	83	9,425,558	53	15	96	11,887,128	58
25	28	1,855,206	10	25	32	2,591,454	12
35	0	0	0	35	16	1,814,180	8

**Table 2:** PM_2.5_ concentrations (un-weighted) (3 year moving average) (in µg/m^3^) in selected cities.

Cities	Population 2010	2002	2003	2004	2005	2006	2007	2008	2009 (Increase)
Riyadh	5,188,286	12.4	13	13.3	13.8	14.8	13.9	15.2	15 (2.6)
Jeddah	3,430,697	17.6	17.2	21.1	20.3	20.6	17.4	18.8	19.4 (1.8)
Makkah	1,534,731	16.5	16.2	20.2	19.1	19.6	16.1	17.6	18.2 (1.7)
Madinah	1,100,093	12.8	12.4	11.6	11.7	11.4	11.1	10.1	10.8 (−2)
Dammam	903,312	27.8	29.6	33.4	30.3	32.5	36	40.4	40 (12.6)
Jubail *	142,825	12.8	13.6	14.6	15.1	15	15.2	15.9	16.4 (3.6)
Yanbu *	73,000	18.9	18.7	19.3	19.1	19.7	21.1	21.3	22.3 (2.4)
Average concentrations (142 cities)	152,675	17.5	17.3	17.9	17.5	18.2	19.1	19.9	20 (2.5)

Note: * Industrial cities.

**Figure 5:**
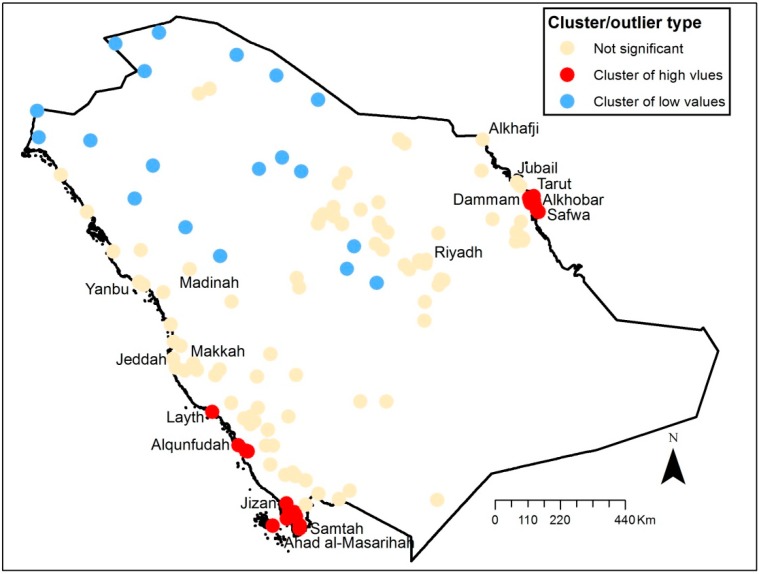
Clusters of PM_2.5_ concentrations (un-weighted) in 2002.

Note: Data Source: Battelle Memorial Institute and CIESIN [1].

**Figure 6:**
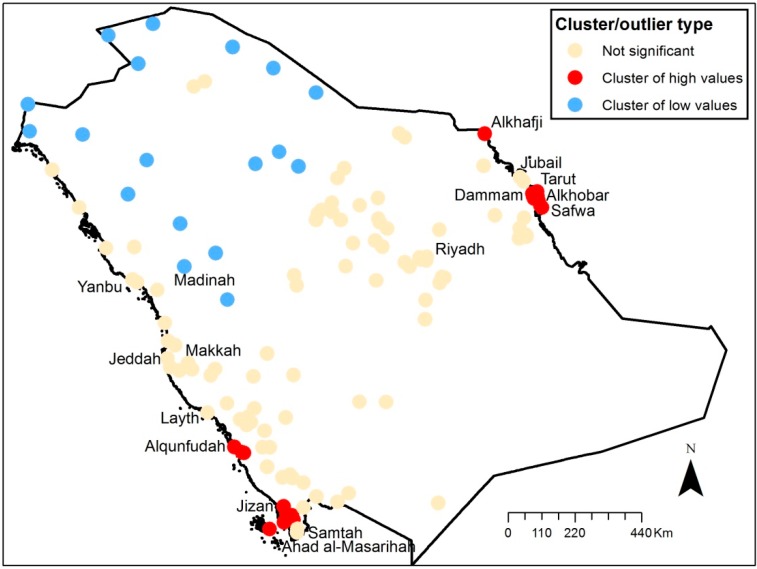
Clusters of PM_2.5_ concentrations (un-weighted) in 2009.

Note: Data Source: Battelle Memorial Institute and CIESIN [1].

Figure 5 and Figure 6 show the results of the clustering analysis performed in ArcGIS. Clusters of low and high values are shown in the maps. The clusters of high values are located in the eastern and south-western part of the country both in 2002 and 2009. The clusters of low values are located in the northern part of Saudi Arabia. For 2002, cities such as Dammam, Tarut, Alkhobar, Safwa, Layth, Alqunfudah, Jizan and Samtah are identified as high value clusters, while Jeddah, Riyadh, Makkah, Madinah, Yanbu and Jubail are identified as “not significant”. For 2009, Alkhafji is identified as having significant cluster of high values, while Samtah, Layth and Ahad Al-Masarihah are no longer considered to have significantly high values. Dammam, Tarut, Alkhobar, Safwa, Alqunfudah and Jizan are still identified as clusters of high values.

## 4. Discussion

The findings of the spatial and temporal analysis of PM_2.5_ concentrations and the cluster analysis have shown a trend whereby the populace is increasingly exposed to fine particulate matter. This trend could be attributed to the increasing anthropogenic activities in Saudi cities. The primary human sources could be vehicle emissions, industries and oil production activities. Saudi Arabia is one of the countries with the highest rates of vehicle ownership due to a lack of public transport systems. The sprawling pattern of the cities, such as Riyadh and Jeddah, also contributes to emissions through long trips to work. Al-Ahmadi and Al-Zahrani [41] noted that NO_2_ concentrations in Jeddah and Riyadh might be caused by vehicle emissions and urban activities, while that of Dammam might be caused by oil production activities. It was also reported that a considerable level of exposure to pollutants was due to traffic emissions in Dammam [42].

The PM_2.5_ concentrations over the major cities like Riyadh and Jeddah are relatively low in comparison with Dammam and Yanbu. However, the large number of people residing in these cities could make it imperative to pay attention to their particulate matter concentrations. Also, the concentrations derived from satellite data might be underestimated. For example, the values reported by Rushdi *et al.* [25] in Riyadh in 2006 and 2007 ranged from 55.6 to 219.5 µg/m^3^. Though the values might be different if annual means are computed from the data, it still shows the possibility of discrepancies between satellite-derived data and ground measurements. Using data collected from 2000–2008 from Saudi AERONET station (Solar Village), Shi *et al.* [43] depicted the correlation coefficients of MISR and MODIS as 0.74 and 0.35 respectively. The values indicate that satellite data can moderately model the PM_2.5_ values over the study area and the level of uncertainty involved. However, it has been noted that increased risk of lung cancer was observed in areas where annual mean levels of PM_2.5_ ranged from 10 to 30 µg/m^3^ [13].

It is noteworthy that the results obtained from this study have some similarity with the outcome of the studies by Al-Ahmadi and Al-Zahrani on NO_2_ and cancer incidence [41] and spatial autocorrelation of cancer incidence [44]. For example, spatial clusters of high incidence of lung cancer were identified in the Eastern Province [44] where there are clusters of high values of PM_2.5_ concentrations. Also, the distribution of mean tropospheric NO_2_ column density showed large concentrations in the Eastern region, Riyadh, Qassim and Makkah [41]. This shows that efforts must be made to improve the monitoring of these pollutants and also to reduce the trend of clustering of high values in some areas especially the Eastern province. As rightly observed by Weng *et al.* [45] that “accurate PM_2.5_ exposure predictions are crucial not only to air quality assessment, but also to address public health concerns”, there is a need to take the advantage of the opportunity presented by satellite-derived data for monitoring.

## 5. Conclusions

This study has used recent population data to reexamine the population-weighted PM_2.5_ values previously computed for Saudi Arabia. It has noted that the values computed by using 2010 population data are higher than the values computed by GRUMP data. It has also highlighted the variations in PM_2.5_ concentrations and exposures values of Saudi Arabian cities. The values for the industrial cities of Jubail and Yanbu are mixed; Yanbu has relatively high values while the values for Jubail are relatively low. Though the resolution of the satellite data is coarse, it still depicts the relative values of the concentrations of PM_2.5_. There is a need for further studies to compute the PM_2.5_ values by using satellite data with higher spatial resolution (similar to the one used by Wong *et al.* [46]) and also calibrate satellite values with ground measurements. The computation of PM_2.5_ values with high resolution data is very relevant at this time that some Saudi cities such as Riyadh and Jeddah are implementing urban observatory to monitor urban growth and its impacts.
